# Investigating the Perceived Efficacy of a Silicone Suturing Task Trainer Using Input from Novice Medical Trainees

**DOI:** 10.7759/cureus.6612

**Published:** 2020-01-09

**Authors:** Patrick O Gallagher, Nicole Bishop, Adam Dubrowski

**Affiliations:** 1 Medicine, Memorial University of Newfoundland, St. John's, CAN; 2 Medical Education and Simulation, Memorial University of Newfoundland, St. John's, CAN; 3 Health Sciences, Ontario Tech University, Oshawa, CAN

**Keywords:** simulation based medical education, three-dimensional printing, suture

## Abstract

Suturing is an essential procedural skill that medical students are expected to be competent in before they graduate medical school; however, there is often a lack of suturing instruction and practice in undergraduate medicine curriculums. Silicone suturing task trainers created from 3D printed molds can help address this gap in medical education by improving student’s manual skills before they perform procedures on real patients upon graduation. Commercially available suture task trainers on the market today lack validation from medical learners; therefore, this study aimed to evaluate the perceived efficacy of a silicone skin suture task trainer created from a 3D printed mold using input from novice medical trainees. A silicone task trainer created by MUN Med 3D was used to teach suturing during two surgery interest group skill development sessions. At the end of the sessions, 38 medical students completed a product evaluation survey that assessed the perceived educational efficacy of the suturing task trainer. Overall, the feedback received from the participants was positive and supported the use of silicone suturing task trainers in undergraduate medicine curriculums.

## Introduction

Simulation-based medical education (SBME) is becoming increasingly popular in healthcare curriculums because it provides a platform for healthcare professionals to acquire necessary procedural skills before performing the procedure on live patients [1-2]. Simulation has been found to increase learner confidence and competence, while also improving patient safety [3]. SBME tools such as those developed through three-dimensional (3D) printing and silicone molding are excellent resources for healthcare learners and instructors since they provide inexpensive, anatomically accurate alternatives to more expensive simulation models [1]. These inexpensive models can provide similar haptic feedback as the more expensive simulators without the fear of high replacement costs [1,4]. However, it is important to note that not all 3D printed models can be created at low cost, especially those that integrate multi-materials and technologies. 

Basic procedural skills, such as suturing and knot tying, require continuous manual practice to develop the necessary hand-eye coordination and dexterity; thus, medical students could benefit from SBME task trainers [5]. Currently, there are two main classes of suturing models found in the literature used for the purpose of suturing simulation: low-fidelity and high-fidelity [5]. Fidelity refers to the “realism” of the model or simulator in comparison to living human patients [6]. Some common low-fidelity models are synthetic skin models (silicone, styrofoam), organic models (orange/banana peels), and various inanimate objects [5-9]. High-fidelity models include animal skin (chicken skin, pig feet) and cadavers [5,10-12]. Although high-fidelity models often provide more realistic simulation to actual patients, there are several drawbacks associated with the use of post-mortem animals and human cadavers. Some of the disadvantages include an increased infection risk, higher costs, storage requirements, and the need for ethics approval [5]. While some advantages to low-fidelity models are that they are relatively inexpensive and portable. Furthermore, low-fidelity simulators allow for unsupervised practice and repeated use [5,13]. Additionally, studies have found that low fidelity models, such as synthetic skin pads, are equally effective as real human tissue to acquire suturing skills [5,14].

When any laceration extends into the subcutaneous tissue, suturing is typically performed to mitigate adverse outcomes during the tissue healing process [15]. The goals of wound closure are to avoid infection and minimize scarring [16]. A proper suturing technique achieves these goals by decreasing the time required for the wound to heal, reducing the likelihood of infection, decreasing the amount of scar tissue formation, and improving cosmetic appearance [15]. However, there is often a lack of instruction on common medical procedures, such as suturing, before performing these techniques on patients [17]. The need for suturing simulation in medical education is evident; however, commercially available silicone suturing task trainers are quite expensive and lack validation from medical professionals and learners [13]. 

This study aims to explore the perceived efficacy of a relatively inexpensive silicone skin suture task trainer created from a 3D printed mold that can be used to train undergraduate medical students. We hypothesize that undergraduate medical students perceive the suturing task trainer to be an efficacious tool to teach suturing in medical school. This research was approved by the local ethics board, which conforms to Code of Ethics of the World Medical Association.

## Materials and methods

Participants

Thirty-nine undergraduate medical students from the Memorial University of Newfoundland (MUN) participated in one, two-hour surgery interest group skill development session. Two of these skills sessions were held (20 students during night one and 19 during night two) in the same week. Of the 39 participants, there were 24 first-year and 14 second-year students (11 second-year on night 1). Of the 39 participants, 38 completed the product evaluation survey. This limitation is likely explained by the voluntary nature of the survey.

Data collection setup

The two skill development sessions, held at MUN Faculty of Medicine multidisciplinary labs, were organized through the MUN Surgery Interest Group. The sessions allowed students to practice various surgical skills under the supervision of a medical educator. The two-hour data collection session consisted of four different skills stations, each occurring in a separate laboratory room. Each station consisted of five participants (four in one station during night two), a research assistant, and a physician educator. All four stations occurred simultaneously, and the groups switched laboratory rooms to practice a different clinical skill every 30 minutes. This research paper only reviews the results from the silicone suturing task trainer. 

**Figure 1:**
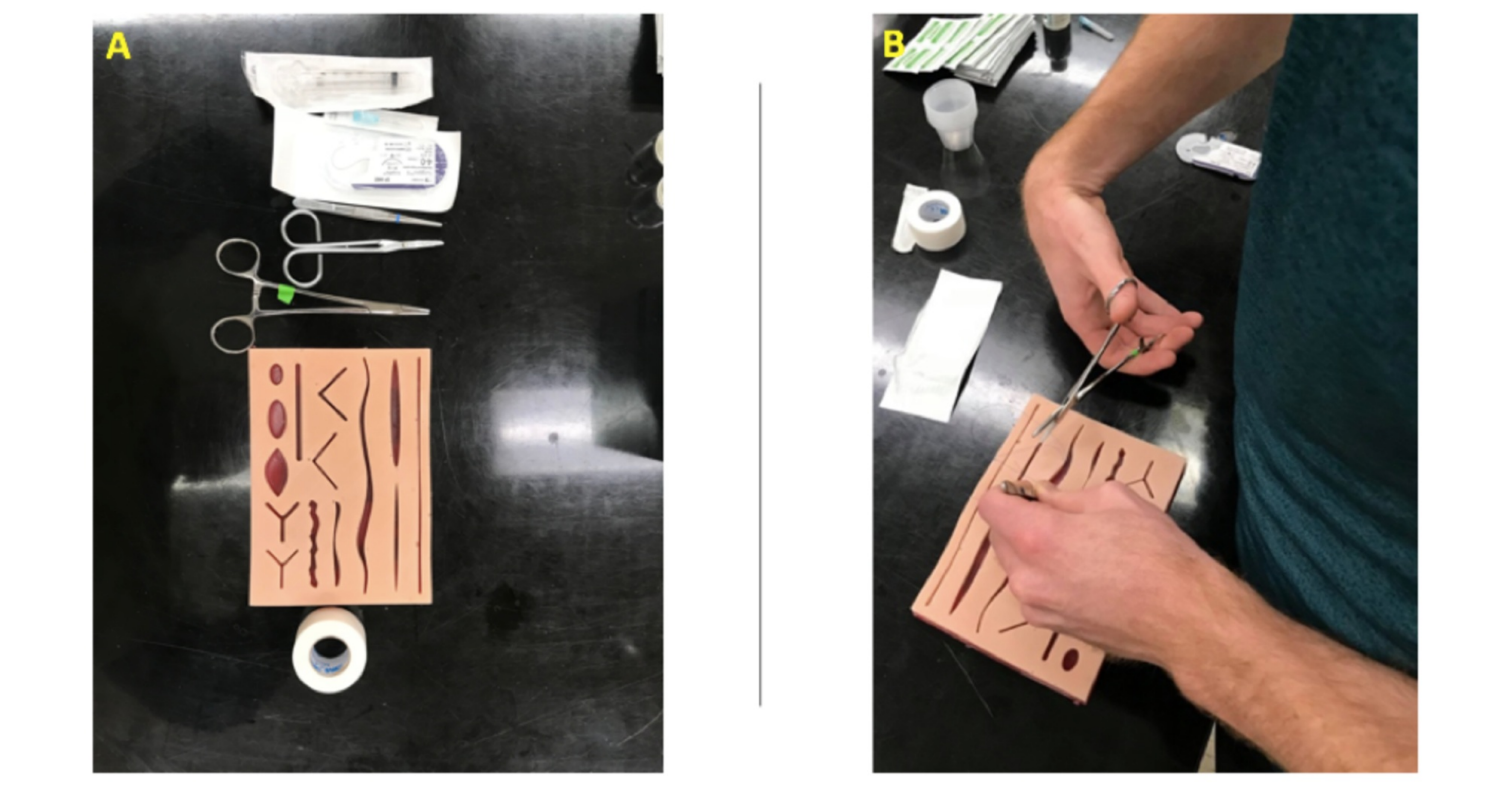
Silicone suturing task trainer used for suturing simulation during the skills night A: Complete set of materials each student was given for a session. B: Student practicing suturing.

The instruments and materials used for the suturing station were identical on both skills nights. Each student was provided with a needle driver, suturing scissors, sutures, surgical tape, anesthetic (lidocaine), skin forceps, latex-gloves, 18-gauge needle, and syringe (Figure 1).

Procedure

Before each skill development session, all students were asked to complete a consent form and provided time to raise any questions or concerns about the study. Each participant then completed a 30-minute suturing stimulation station where they received approximately 10 minutes of instruction from a physician educator and 15 minutes to practice their suturing technique using the suture skin task trainer. The remaining 5 minutes at each station was designated for students to complete the product evaluation surveys on the specific task trainer used for each station.

Although the instructors were different for each skill development session (emergency medicine physician on night one and a second-year general surgery resident on night two), they both taught the same chronological steps for proper wound closure. Both educators began by explaining the importance of sterility, patient preparation, and steps to properly clean the wound. Furthermore, the instructors taught simple interrupted and horizontal mattress suture techniques using the silicone suture skin task trainer in a stepwise fashion according to Newell (2007). The task trainer was also used to demonstrate how local anesthetic is applied to a wound before closure. Following the demonstration, each student was given an identical task trainer and set of instruments to practice individually. During the 15-minute practice period, the instructors remained at the station to answer questions and provide additional guidance. In the last 5 minutes of the 30-minute suturing station, the research assistant provided a brief survey that evaluated the suturing-task trainer.

Data Collection Instrument

The product evaluation survey used to determine the efficacy of the suture task trainer as an educational tool consisted of 18 questions. Only 11 questions were included in this report (Q2-11, 18) because many of the undergraduate students had limited suturing practice and few had sutured on real patients. Therefore, the survey data corresponding to the realism of the task trainer was excluded from the results. The evaluation survey created for this study assessed the task trainer through both quantitative and qualitative measures. The four quantitative questions on the survey required participants to score the educational efficacy of the task trainer on a 5-point Likert scale. The six qualitative measures consisted of two opened-ended questions prompting feedback about the model or simulation experience and four close-ended “yes or no” questions that assessed model efficacy and participant’s previous suturing experience. The survey also included one 3-point global rating scale that assessed how many improvements, if any, need to be made to the task trainer. 

Statistical Analysis

All responses to the 5-point Likert scale questions in the survey were analyzed using SPSSTM software (IBM Corp. Released 2017. IBM SPSS Statistics for Mac, Version 25.0. Armonk, NY: IBM Corp.). A Shaprio-Wilk test was performed on all response data to determine normality as there was a small sample size (<50 samples) [18]. The data from the study were not normally distributed. Therefore, a Mann-Whitney U test was used to determine if there were significant differences in responses between the two skills nights. For all statistical tests, significance cut-off points were applied as p-values of ≤0.05.

## Results

Quantitative

The aim of this results section is to present the data that is relevant to the educational application of the silicone suturing task trainer. The Mann-Whitney U test revealed that there was no significant difference in responses between the two skills nights; therefore, it is unlikely that the different instructors and uneven distribution of first and second-year students, influenced the participant’s survey responses.

**Figure 2:**
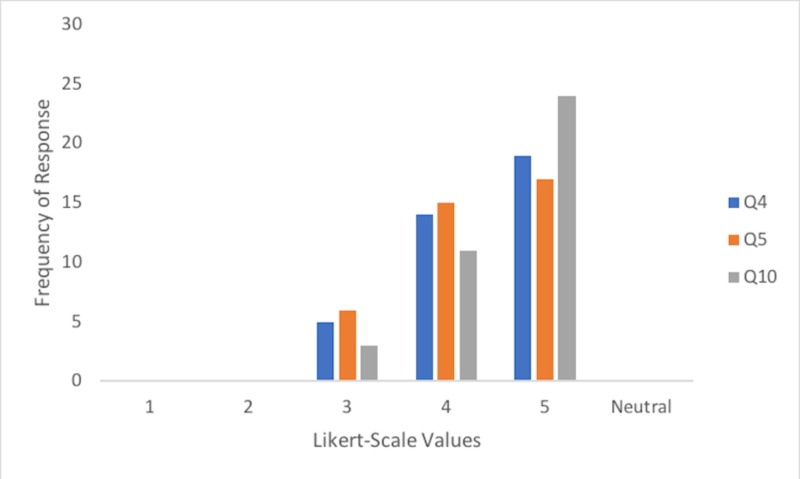
Frequency of response for suture task trainer product evaluation survey questions 4, 5 and 10 (n = 38) Q4: How effective is the suture skin task trainer in increasing your competency in suturing?  Q5: How effective is the suture skin task trainer in increasing your confidence to perform suturing? Q10: Rate the value of the suture skin task trainer as a training tool.

Overall, the survey results received from undergraduate medical students during the skills nights were positive and supported the use of silicone suturing task trainers from a 3D printed mold. Thirty-eight of the thirty-nine students that practiced suturing on the task trainer completed the entire survey (one student chose to not fill out the survey). Students found the suture task trainer increased their suturing competence and confidence (Figure 2). Furthermore, the majority of participants found the suture task trainer was a valuable training tool (Figure 2).

Qualitative

There was an open-ended question in the survey that asked for “additional comments”. Some notable responses include: “too much tension in skin when closing the wound”, “material is too easy to go through”, and “material should be more pliable so that wound joins together like real skin”. Many students also indicated that they enjoyed the “various sizes and shapes” of the lacerations because they could practice various suturing strategies. Furthermore, from previous suturing experiences, students noted that they felt that the silicone task trainer was more realistic than the “high-fidelity” pig feet.

**Figure 3:**
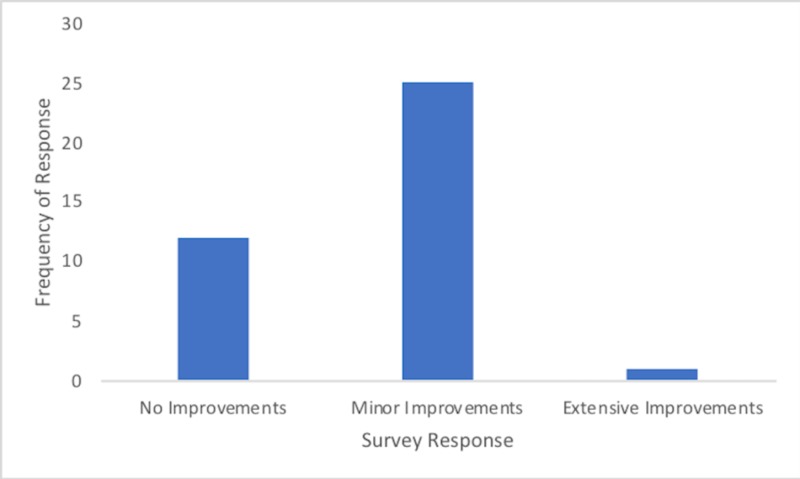
Frequency of responses for the global rating of improvements required for the suturing skin task trainer (n = 38)

Thirty-one percent of students felt that no improvements should be made to the suture skin task trainer, while 66% felt minor improvements are required and 3% felt that extensive improvements should be made to the suturing skin task trainer (Figure 3). The improvements suggested by the study participants included creating a more distinct boundary between the epidermis and dermis, making the skin more elastic so that wounds can close more tightly, and adding more wound types. Additionally, 100% of the study participants felt that the suture skin task trainer helped them understand something about suturing they did not previously. All participants also indicated that they would like to use the suture skin task trainer throughout their medical training and they would recommend it to other medical students to assist in their education.

## Discussion

The silicone skin suture task trainer product evaluation survey provided excellent feedback to gauge if undergraduate medical students found the model to be a valuable teaching tool. Overall, the results of this study suggest that the suturing task trainer is a highly efficacious simulation model to teach suturing in undergraduate medical education.

Most students indicated that they appreciated the various sizes and shapes of the lacerations on the suturing skin task trainer. The different lacerations allowed the students to practice a variety of wound closure strategies required to close a particular wound type. Highly realistic simulators, such as those based on animal skin, do not have different laceration shapes and sizes, which are valuable to help teach different wound closure approaches. It is noteworthy that each suture skin task trainer was in excellent condition after eight students practiced suturing for 15 minutes on it. Therefore, the durability of the silicone suture skin task trainer supports repeated use, which contributes to the cost efficiency over animal simulators or human cadavers.

While the survey feedback was positive overall and supported the use of the suture skin task trainer, this study also indicates that there are a few aspects that can be improved upon in future iterations of this simulator. Many of the survey open-ended responses and verbal comments from participants during the session mentioned that the silicone material was too stiff and not elastic enough to bring the wound edges together when suturing. These comments were also supported by feedback from the emergency medicine physician and general surgery resident that instructed the sessions.

Now that the silicone task trainer has been found to be an efficacious training tool, the next steps in this investigation will focus on face and content validity from experts (i.e. realism and educational efficacy), and finally assessment of two aspects of construct validity: different performances by different levels of expertise, and sensitivity to change due to repetitive practice by novice learners.

## Conclusions

Before this study, there were no studies that examined the value of silicone suturing task trainers as an educational tool. However, the responses from novice medical trainees in this study support the use of silicone skin task trainers in medical education. After incorporating improvement suggestions received from this study and evaluating face and content validity from suturing experts, this suture skin task trainer can be used in undergraduate medical education curriculums. Since suturing requires continuous practice to develop proper technique and dexterity, it is evident that medical learners could benefit greatly from this easily portable, relatively low-cost suturing task trainer.
